# Synthesis and Anticancer Evaluation of Some New 3-Benzyl-4,8-Dimethylbenzopyrone Derivatives

**DOI:** 10.2174/1874104501711010081

**Published:** 2017-09-21

**Authors:** Sohair L. El-Ansary, Doaa E. Abdel Rahman, Lina M. A. Abdel Ghany

**Affiliations:** 1 Department of Pharmaceutical Chemistry, Faculty of Pharmacy, Cairo University, Kasr El-Aini Street, Cairo 11562, Egypt; 2 Department of Pharmaceutical Chemistry, Faculty of Pharmacy, Misr University for Science and Technology, Misr, Egypt

**Keywords:** Benzopyrones, Acid hydrazide, Oxadiazoles, Pyrazoles, Anticancer

## Abstract

**Introduction:**

New benzopyrone derivatives such as Schiff’s like compounds, acetohydrazides or substituted with oxadiazole or pyrazole heterocycles were synthesized from parent acid hydrazide compound **3**.

**Methods and Materials:**

Structures of the synthesized compounds were elucidated using IR, NMR and mass spectroscopy. All the synthesized derivatives were selected by National Cancer Institute (NCI), Bethesda, and evaluated for their *in vitro* anticancer activity in the full NCI 60 cell lines panel assay.

**Results and Conclusion:**

Schiffs like compounds **4a, b** and **c** were found to have good growth inhibition % against numerous cell lines that belong mainly to leukemia, non-small cell lung, CNS and breast Cancer subpanels.

## 1 INTRODUCTION

Cancer can be defined as a disease in which a group of abnormal cells grow uncontrollably disregarding the normal rules of cell division. Normal cells are constantly subjected to signals that dictate whether the cell should divide, differentiate into another cell or die. Cancer cells develop a degree of autonomy from these signals, resulting in uncontrolled growth and proliferation. If this proliferation is allowed to continue and spread, it can be fatal. In fact, almost 90% of cancer- related deaths are due to tumor spreading or dissemination [1]. Phenomenal advances in cancer research have given us insight into how cancer cells develop this autonomy. Now, cancer is defined as a disease that involves changes or mutations in the cell genom, the somatic mutation theory has been the prevailing paradigm in cancer research and its premise is that cancer is a disease of cell proliferation caused by mutation in genes that control proliferation and the cell cycle [2].

Although advances in the field of chemo-preventive and therapeutic medicine have been made regularly over the last ten years, the search for novel anticancer treatments continues as it became an urgency to develop new anticancer agents with fewer side effects.

**Fig. (1):**
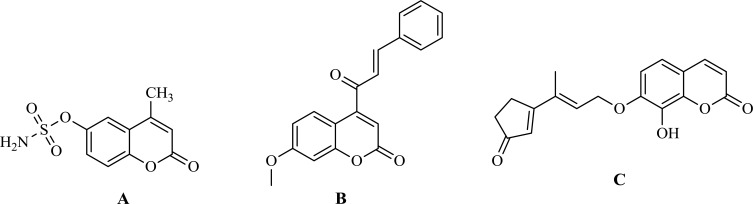
Antitumor benzopyrones.

Benzopyran-2-one comprises a group of natural compounds found in a variety of plant sources [3]. Benzopyrones were recognized to possess a broad spectrum of antitumor activity following different mechanisms as 667-Coumarate (Fig. **1A**) that acts as Sulphatase inhibitors [4] and Carbonic anhydrase II enzyme inhibitors [5] while other benzopyrones were reported as Histone deacetylase (HDAC) inhibitors as Fig. (**1B**) [6]. Moreover, geiparvarin (Fig. **1C**), a naturally occurring coumarin has been shown to possess a significant inhibition for cell lines including sarcoma 180, lewis lung carcinoma, P-388 lymphocytic leukemia and walker 256 carcinosarcoma [7]. Furthermore, literature survey revealed several heterocycles as oxadiazole [8], pyrazole [9], dimethyl pyrazole [10], amino pyrazole [11], and pyrazolone [12] all possessed reported antitumor effect.

These findings have encouraged us to design and synthesize compounds comprised of the benzopyran-2-one scaffold as Schiffs like compounds, acetohydrazides or substituted with oxadiazole, pyrazole heterocycles. The newly synthesized compounds were selected by National Cancer Institute (NCI), Bethesda, MD, U.S.A., for *in vitro* one dose testing in the full NCI 60 cell lines panel assay.

## 2 MATERIALS AND METHODS

### 2.1 Chemistry

Melting points were determined by open capillary tube method using Stuart SMP10 melting point apparatus and were uncorrected. Microanalyses were carried out at The Regional Center for Mycology and Biotechnology, Al-Azhar University. Infrared Spectra were recorded as potassium bromide discs on Schimadzu FT-IR 8400S spectrophotometer (Shimadzu, Kyoto, Japan) and Bruker FT-IR spectrophotometer and expressed in wave number υ_max_ (cm^-1^). The ^1^H NMR spectra were recorded on a Bruker AVANCE III spectrometer at 400 MHz, in dimethylsulphoxide (DMSO-*d_6_*). Chemical Shifts are quoted in δ as parts per million (ppm) downfield from tetramethylsilane (TMS) as internal standard and *J* values are reported in Hz. Mass spectra were performed as EI at 70eV on Hewlett Packard Varian (Varian, Polo, USA) and Shimadzu Gas Chromatograph Mass spectrometer-QP 1000 EX and direct inlet unit of Shimadzu GC/MS-QP5050A at 70eV. TLC were carried out using Macherey-Nagel Alugram Sil G/UV_254_ silica gel plates with fluorescent indicator UV_254_ and chloroform:methanol (9.5:0.5) as the eluting system and the spots were visualized at 366, 254 nm by UV Vilber Lourmat 77202 (Vilber, Marne La Vallee, France).

#### 2.1.1 **3-Benzyl-4,8-dimethyl-7-hydroxy-2*H*-1-benzopyran-2-one 1 (Scheme **1**)** was prepared as reported in literature [13].

#### 2.1.2 Ethyl 2-(3-benzyl-4,8-dimethyl-2-oxo-2*H*-benzopyran-7-yl)oxyacetate 2 (Scheme **1**).

A mixture of **1** (28 g, 0.1 mol), anhydrous potassium carbonate (27.6 g, 0.2 mol) and ethyl chloroacetate (14.64 g, 0.12 mol) in dry acetone (200 mL) was heated under reflux with stirring for 24 h. It was then made to cool down filtered and washed with acetone. The combined filtrate and washing were concentrated and filtered. The crude product was crystallized from ethanol to yield 36% of **2**. mp 103-105 ^o^C. IR υ_max,_ cm^-1^: 3010 (CH Ar), 2916 (CH aliphatic), 1705, 1685 (2C=O), 1602, 1577, 1492, (C=C). ^1^H NMR (DMSO-*d_6_*) δ *ppm*: 1.22 (t, 3H, CH_2_CH_3_), 2.25 (s, 3H, CH_3_ at C4), 2.42 (s, 3H, CH_3_ at C8), 3.96 (s, 2H, CH_2_), 4.18 (q, 2H, CH_2_CH_3_), 4.96 (s, 2H, OCH_2_), 6.98 (d, 1H, *J*=9.0 Hz, H-6 Ar), 7.17 (t, 1H, H-4′ Ar), 7.23 (t, 2H, H-3′,5′ Ar), 7.28 (d, 2H, *J* = 7.4 Hz, H-2′,6′ Ar), 7.62 (d, 1H, J=8.9 Hz, H-5 Ar). MS m/z %: 366 (M^+^) 100%. C_22_H_22_O_5_ (366.41): Anal. Calcd. for Calc. C, 72.12; H, 6.05. Found: C, 72.48; H, 6.17.

#### 2.1.3 2-(3-Benzyl-4,8-dimethyl-2-oxo-2*H*-benzopyran-7-yl)oxyacetohydrazide 3 (Scheme **1**)

A mixture of the ester compound **2** (3.66 g, 0.01 mol) and hydrazine hydrate 99% (1 mL, 0.02 mol) in ethanol (30 mL) was heated under reflux for 2 h. The precipitate was filtered, washed with water and dried. The crude product was crystallized from acetic acid to yield 89% of **3**. mp 243-245^o^C. IR υ_max,_ cm^-1^: 3502, 3446, 3180 broad (NH_2_, NH), 3061 (CH Ar), 2929, 2854 (CH aliphatic), 1705, 1697 (2C=O), 1604, 1495 (NH, C=C). ^1^H NMR (DMSO-*d_6_*) δ *ppm*: 2.26 (s, 3H, CH_3_ at C4), 2.43 (s, 3H, CH_3_ at C8), 3.96 (s, 2H, CH_2_), 4.35 (br’s, 2H, NH_2_), 4.65 (s, 2H, OCH_2_), 6.96 (d, 1H, *J*=9.0 Hz, H-6 Ar), 7.17 (t, 1H, H-4′ Ar), 7.23 (t, 2H, H-3′,5′ Ar), 7.28 (d, 2H, *J* = 7.4 Hz, H-2′,6′ Ar), 7.63 (d, 1H, J=8.92 Hz, H-5 Ar), 9.32 (s, 1H, NH). MS m/z %:352 (M^+^) 75.48%. Anal. Calcd. for C_20_H_20_N_2_O_4_ (352.38): Calc.: C, 68.17; H, 5.72; N, 7.95. Found: C, 68.34; H, 5.87; N, 8.10.

#### 2.1.4 General procedure for synthesis of 2-(3-Benzyl-4,8-dimethyl-2-oxo-2*H*-benzopyran-7-yl)oxy-*N*′-(Substitutedbenzylidene)acetohydrazide 4a-d (Scheme **1**)

A mixture of acid hydrazide **3** (0.01 mol), appropriate aromatic aldehyde/acetophenone (0.01 mol) in ethanol (20 mL) containing a few drops of acetic acid was heated under reflux for 18-24 h. The solvent was distilled under vacuum and the residue crystallized from ethanol.

##### 2.1.4.1 2-(3-Benzyl-4,8-dimethyl-2-oxo-2*H*-benzopyran-7-yl)oxy-*N*′-(4-dimethylaminobenzylidene)acetohydrazide 4a

Yield 25%. mp 64-67 °C. IR υ_max_/ cm^-1^: 3460 (NH), 3057 (CH Ar), 2926 (CH aliphatic), 1710, 1693, (2C=O), 1600, 1643, 1554, 1494, (C=N, NH, C=C). ^1^H NMR (DMSO-*d_6_*) δ *ppm*: 2.26 (s, 3H, CH_3_ at C4), 2.42 (s, 3H, CH_3_ at C8), 3.07 (s, 6H, N(CH_3_)_2_), 3.96 (s, 2H, CH_2_), 5.28 (s, 2H, OCH_2_), 6.72 (d, 1H, *J* = 8.8 Hz, H-6 Ar), 6.79 (d, 2H, *J* = 8.9 Hz, H-3′′,5′′ Ar), 7.17 (t, 1H, H-4′ Ar), 7.23 (t, 2H, H-3′,5′ Ar), 7.26 (d, 2H, *J* = 7.4 Hz, H-2′,6′ Ar), 7.51 (d, 1H, *J* = 8.7 Hz, H-5 Ar), 7.69 (d, 2H, *J* = 8.9 Hz, H-2′′,6′′ Ar), 7.89 (c, 1H, CH=N), 8.50 (s, 1H, NH). MS *m/z*%: 483 (M^+^) 11.33%. Anal. Calcd. For C_29_H_29_N_3_O_4_ (483.56): C, 72.03; H, 6.04; N, 8.69 Found: C, 72.19; H, 6.11; N, 8.85.

##### 2.1.4.2 2-(3-Benzyl-4,8-dimethyl-2-oxo-2*H*-benzopyran-7-yl)oxy-*N*′-(4-methoxybenzylidene)acetohydrazide 4b

Yield 71%. mp 221-223 °C. IR υ_max_/ cm^-1^: 3432 (NH), 3090 (CH Ar), 2967, 2927 (CH aliphatic), 1713 (2C=O), 1614, 1449 (C=N, C=C). ^1^H NMR (400 MHz, DMSO-*d_6_*) δ *ppm*: 2.27 (s, 3H, CH_3_ at C4), 2.42 (s, 3H, CH_3_ at C8), 3.80 (s, 3H, OCH_3_), 3.96 (s, 2H, CH_2_), 5.30 (s, 2H, OCH_2_), 6.96 (d, 1H, *J* = 9.1 Hz, H-6 Ar), 6.99 (d, 2H, *J* = 8.7 Hz, H-3′′,5′′ Ar), 7.17 (t, 1H, H-4′ Ar), 7.23 (t, 2H, H-3′,5′ Ar), 7.26 (d, 2H, *J* = 7.4 Hz, H-2′,6′ Ar), 7.60 (d, 1H, *J* = 8.9 Hz, H-5 Ar), 7.69 (d, 2H, *J* = 8.7 Hz, H-2′′,6′′ Ar),7.96 (c, 1H, CH=N), 11.54 (s, 1H, NH). MS *m/z*%: 469 (M^+^-1) 89.14%, 470 (M^+^) 29.32%. Anal. Calcd. For C_28_H_26_N_2_O_5_ (470.52): C, 71.47; H, 5.57; N, 5.95. Found: C, 71.80; H, 5.64; N, 6.04.

##### 2.1.4.3 2-(3-Benzyl-4,8-dimethyl-2-oxo-2*H*-benzopyran-7-yl)oxy-*N*′-(3,4,5-trimethoxybenzylidene)acetohydrazide 4c

Yield 86%. mp 140-142 °C. IR υ_max_/ cm^-1^: 3203 (NH), 3062 (CH Ar), 2958, 2924 (CH aliphatic), 1695, 1681 (2C=O), 1602, 1566, 1508, 1492 (C=N, NH, C=C). ^1^H NMR (400 MHz, DMSO-*d_6_*) δ *ppm*: 2.27 (s, 3H, CH_3_ at C4), 2.42 (s, 3H, CH_3_ at C8), 3.70 (s, 3H, OCH_3_), 3.82 (s, 6H, 2xOCH_3_), 3.97 (s, 2H, CH_2_), 5.35 (s, 2H, OCH_2_), 6.96 (d, 1H, J=8.9 Hz,H-6 Ar), 7.01 (s, 2H, H-2′′,6′′ Ar), 7.17 (t, 1H, H-4′ Ar), 7.23 (t, 2H, H-3′,5′ Ar), 7.26 (d, 2H, *J* = 7.4 Hz, H-2′,6′ Ar), 7.62 (d, 1H, *J* = 8.8 Hz, H-5 Ar), 8.22 (s, 1H, HC=N), 11.57 (s, 1H, NH). MS *m/z*%: 530 (M^+^) 34.00%. Anal. Calcd. for C_30_H_30_N_2_O_7_ (530.57): C, 67.91; H, 5.70; N, 5.28 Found: C, 68.17; H, 5.79; N, 5.39.

##### 2.1.4.4 2-(3-Benzyl-4,8-dimethyl-2-oxo-2*H*-benzopyran-7-yl)oxy-*N*′-[1- (3,4-dimethoxyphenyl) ethylidene] acetohydrazide 4d

Yield 30%. mp 273-275 °C. IR υ_max_/ cm^-1^: 3188 (NH), 3059 (CH Ar), 2931, 2852 (CH aliphatic), 1710, 1697 (2C=O), 1604, 1514, 1490, (C=N, C=C). ^1^H NMR (DMSO-*d_6_*) δ *ppm*: 2.25 (s, 3H, CH_3_ at C4), 2.42 (s, 3H, CH_3_ at C8), 2.53 (s, 3H, N=CCH_3_), 3.81 (s, 3H, OCH_3_), 3.84 (s, 3H, OCH_3_), 3.96 (s, 2H, CH_2_), 4.96 (s, 2H, OCH_2_), 6.97 (d, 1H, *J* = 8.8 Hz, H-6 Ar), 7.06 (d, 1H, *J* = 8.4 Hz, H-6′′Ar), 7.17 (t, 1H, H-4′ Ar), 7.23 (t, 2H, H-3′,5′ Ar), 7.27 (d, 2H, *J* = 7.4 Hz, H-2′,6′ Ar), 7.44 (d, 1H, *J* = 5.9 Hz, H-2′′ Ar), 7.60-7.64 (m, 3H, H-5,5′′Ar, NH). MS m/z %: 514 (M^+^) 1.20%. Anal. Calcd. for C_30_H_30_N_2_O_6_ (514.57): C, 70.02; H, 5.88; N, 5.44. Found: C, 70.38; H, 5.94; N, 5.60.

#### 2.1.5 *N*′-Acetyl-2-(3-benzyl-4,8-dimethyl-2-oxo-2*H*-benzopyran-7-yl)oxyacetohydrazide 5 (Scheme **1**)

A suspension of acid hydrazide **3** (1.83 g, 0.005 mol) in glacial acetic acid (15 mL) was stirred at room temperature for 24 h. The solvent was distilled under vacuum and the residue was crystallized from ethyl acetate to yield 98% of **5**. mp 278-279 ºC. IR υ_max,_ cm^-1^: 3446, 3238 (2 NH), 3001 (CH Ar), 2922 (CH aliphatic), 1715, 1707 (3 C=O), 1653, 1602, 1583, 1487 (NH, C=C). ^1^H NMR (DMSO-*d_6_*) δ *ppm*: 1.88 (s, 3H, COCH_3_), 2.24 (s, 3H, CH_3_ at C4), 2.43 (s, 3H, CH_3_ at C8), 3.96 (s, 2H, CH_2_), 4.76 (s, 2H, OCH_2_), 7.00 (d, 1H, *J*=9.0 Hz, H-6 Ar), 7.17 (t, 1H, H-4′ Ar), 7.23 (t, 2H, H-3′,5′ Ar), 7.28 (d, 2H, *J* = 7.4 Hz, H-2′,6′ Ar),7.63 (s, 1H, *J*=8.8 Hz, H-5 Ar), 9.89 (s, 1H, NH), 10.04 (s, 1H, NH). MS m/z %:394 (M^+^) 100%. Anal. Calcd. for C_22_H_22_N_2_O_5_ (394.42): Calc.: C, 66.99; H, 5.62; N, 7.10. Found: C, 67.26; H, 5.71; N, 7.23.

#### 2.1.6 3-Benzyl-4,8-dimethyl-7-(5-methyl-1,3,4-oxadiazol-2-yl)methoxy-2*H*-benzopyran-2-one 6 (Scheme **1**)

A mixture of compound **5** (1.97 g, 0.005 mol) and phosphorus oxychloride (3 mL) in dioxane (10 mL), was heated under reflux for 3 h. The reaction mixture was cooled down, diluted with ice-cold water and neutralized with ammonium hydroxide. The precipitate formed was filtered, dried and crystallized from ethanol to yield 63% of **6**. mp >350 ºC. IR υ_max,_ cm^-1^: 3061 (CH Ar), 2900, 2852 (CH aliphatic), 1691(C=O), 1624, 1610, 1490 (C=N, C=C). ^1^H NMR (DMSO-*d_6_*) δ *ppm*: 1.87 (s, 3H, CH_3_), 2.27 (s, 3H, CH_3_ at C4), 2.43 (s, 3H, CH_3_ at C8), 3.96 (s, 2H, CH_2_), 4.75 (s, 2H, OCH_2_), 7.02 (d, 1H, *J*=8.9 Hz, H-6 Ar), 7.17 (t, 1H, H-4′ Ar), 7.23 (t, 2H, H-3′,5′ Ar), 7.28 (d, 2H, *J* = 7.4 Hz, H-2′,6′ Ar), 7.63 (s, 1H, *J*=8.8 Hz, H-5 Ar). MS m/z %: 376 (M^+^) 100%. Anal. Calcd. for C_22_H_20_N_2_O_4_ (376.41): Calc.: C, 70.20; H, 5.36; N, 7.44. Found: C, 70.39; H, 5.44; N, 7.68.

#### 2.1.7 2-(3-Benzyl-4,8-dimethyl-2-oxo-2*H*-benzopyran-7-yl)oxy-*N*,*N*′-bis(4-methylphenylsulfonyl)acetohydrazide 7 (Scheme **1**)

A mixture of acid hydrazide **3** (1.83 g, 0.005mol), tosyl chloride (1.06 g, 0.01 mol) in ethanol (20 mL) containing a few drops of acetic acid was heated under reflux for 12 h. The solvent was distilled under vacuum and the residue crystallized from ethanol to yield 35% of **7**. mp 200-203 ºC. IR υ_max,_ cm^-1^:3446 (NH), 3032 (CH Ar), 2920, 2864 (CH aliphatic), 1743, 1705 (2C=O), 1602, 1496, (NH, C=C), 1354, 1188 (2 SO_2_). ^1^H NMR (DMSO-*d_6_*) δ *ppm*: 2.24 (s, 3H, CH_3_ at C4), 2.29 (s, 6H, 2xCH_3_), 2.42 (s, 3H, CH_3_ at C8), 3.96 (s, 2H, CH_2_), 4.00 (br s, 1H, NH, exchanged with D_2_O), 4.86 (s, 2H, OCH_2_), 6.96 (d, 1H, *J*=9.0 Hz, H-6 Ar), 7.12 (d, 4H, *J*=8.0 Hz, 2xH-3ʺ,5ʺ Ar), 7.17 (t, 1H, H-4′ Ar), 7.23 (t, 2H, H-3′,5′ Ar), 7.27 (d, 2H, *J*=7.4 Hz, H-2′,6′ Ar), 7.50 (d, 4H, *J*=8.0 Hz, 2xH-2ʺ,6ʺ Ar), 7.62 (d, 1H, *J*=9.0 Hz, Hz, H-5 Ar). MS m/z %: 660 (M^+^) 0.55%. Anal. Calcd. for C_34_H_32_N_2_O_8_S_2_ (660.65): Calc.: C, 61.80; H, 4.88; N, 4.24. Found: C, 61.48; H, 4.96; N, 4.41.

#### 2.1.8 7-[2-(3-Amino-5-imino-4,5-dihydropyrazol-1-yl)-2-oxoethoxy]-3-benzyl-4,8-dimethyl-2*H*-benzopyran-2-one 8 (Scheme **2**)

A mixture of the hydrazide compound **3** (1.83 g, 0.005mol) and malononitrile (0.66 g, 0.01 mol) in ethanol (15 mL) was heated under reflux for 18h. The formed precipitate was filtered, washed with water, dried and crystallized from acetic acid to yield 89% of **8**. mp 253-255^o^C. IR υ_max_/ cm^-1^: 3504, 3448, 3404 (NH_2_, NH), 3022 (CH Ar), 2933 (CH aliphatic), 1695, 1678 (2C=O), 1627, 1514, 1452 (NH, C=C). ^1^H NMR (DMSO-*d_6_*) δ *ppm*: 1.88 (s, 2H, CH_2_ pyrazoline), 2.27 (s, 3H, CH_3_ at C4), 2.43 (s, 3H, CH_3_ at C8), 3.97 (s, 2H, CH_2_) 4.65 (s, 1H, NH), 4.77 (s, 2H, OCH_2_), 7.00 (d, 1H, *J*=8.0 Hz, H-6 Ar), 7.18 (t, 1H, H-4′ Ar), 7.23 (t, 2H, H-3′,5′ Ar), 7.28 (d, 2H, *J* = 7.4 Hz, H-2′,6′ Ar), 7.64 (d, 1H, *J*=8.0 Hz, H-5 Ar), 9.93 (s, 2H, NH_2_). MS m/z%: 420 (M^+^+2) 2.88%. Anal. Calcd. for C_23_H_22_N_4_O_4_ (418.45): C, 66.02; H, 5.30; N, 13.39. Found: C, 66.34; H, 5.35; N, 13.64.

#### 2.1.9 1-[2-(3-Benzyl-4,8-dimethyl-2-oxo-2*H*-benzopyran-7-yl)oxyacetyl]-5-iminopyrazolidin-3-one 9 (Scheme **2**)

A mixture of the acid hydrazide **3** (1.83 g, 0.005 mol) and ethyl cyanoacetate (1.13 mL, 0.01 mol) in ethanol (5 mL) was heated under reflux for 6 h. The precipitated solid was filtered, washed, dried and crystallized from ethanol to yield 65% of **9**. mp 215-217 ^o^C. IR υ_max,_ cm^-1^: 3446, 3176 (2NH), 3059 (CH Ar), 2920, 2840 (CH aliphatic), 1710, 1697 (3C=O), 1620, 1606, 1492 (NH, C=C). ^1^H NMR (DMSO-*d_6_*) δ *ppm*: 1.87 (s, 2H, CH_2_ pyrazolone), 2.26 (s, 3H, CH_3_ at C4), 2.43 (s, 3H, CH_3_ at C8), 3.96 (s, 2H, CH_2_), 4.76 (s, 2H, OCH_2_), 7.00 (d, 1H, *J*= 8.9 Hz, H-6 Ar), 7.17 (t, 1H, H-4′ Ar), 7.23 (t, 2H, H-3′,5′ Ar), 7.28 (d, 2H, *J* = 7.4 Hz, H-2′,6′ Ar), 7.63 (d, 1H, *J*=8.9 Hz, H-5 Ar), 9.67 (s, 2H, 2xNH). MS m/z %:419 (M^+^) 1.18%. Anal. Calcd. for C_23_H_21_N_3_O_5_ (419.43): Calc.: C, 65.86; H, 5.05; N, 10.02. Found: C, 66.14; H, 5.19; N, 10.31.

#### 2.1.10 1-[2-(3-benzyl-4,8-dimethyl-2-oxo-2*H*-benzopyran-7-yl)oxyacetyl]-5-imino-2,5-dihydro-1*H*-pyrazole-4-carbonitrile 10 (Scheme **2**)

A mixture of the acid hydrazide **3** (1.83 g, 0.005 mol) and ethoxy methylene malononitrile (1.22 g, 0.01 mol) in ethanol (15 mL) was heated under reflux for 24 h. The solution was concentrated and the precipitated solid was filtered, washed, dried and crystallized from DMF to yield 45% of **10**. mp 200-205 ^o^C. IR υ_max,_ cm^-1^: 3421, 3367 (2NH), 2929, 2856 (CH aliphatic), 2196 (CN), 1700, 1680 (2 C=O), 1622, 1602, 1544, 1492 (C=N, NH, C=C). ^1^H NMR (DMSO-*d_6_*) δ *ppm*: 2.25 (s, 3H, CH_3_ at C4), 2.42 (s, 3H, CH_3_ at C8), 3.96 (s, 2H, CH_2_), 4.65 (s, 2H, OCH_2_), 6.95 (d, 1H, *J*=8.9 Hz, H-6 Ar), 7.15-7.28 (m, 6H, H-Ar, CH pyrazole), 7.62 (d, 1H, *J*=8.9 Hz, H-5 Ar), 8.62 (s, 1H, NH exchanged with D_2_O), 9.34 (s, 1H, NH exchanged with D_2_O). MS m/z %:427 (M^+^-1) 6.04%. Anal. Calcd. for C_24_H_20_N_4_O_4_ (428.44): Calc.: C, 67.28; H, 4.71; N, 13.08. Found: C, 67.51; H, 4.80; N, 13.25.

#### 2.1.11 3-Benzyl-7-[2-(3,5-dimethyl-1*H*-pyrazol-1-yl)-2-oxoethoxy]-4,8-dimethyl-2*H*-benzopyran-2-one 11 (Scheme **2**)

A mixture of the acid hydrazide **3** (1.83 g, 0.005 mol) and acetyl acetone (1 mL) in ethanol containing a few drops of triethylamine was heated under reflux for 24 h. The solution was concentrated and the precipitated solid was filtered, washed, dried and crystallized from DMF to yield 45% of **7**. mp 100-101^o^C. IR υ_max,_ cm^-1^: 3030 (CH Ar), 2926, 2858 (CH aliphatic), 1743, 1705 (2C=O), 1602, 1498 (C=C). ^1^H NMR (DMSO-*d_6_*) δ *ppm*: 2.26 (s, 3H, CH_3_ at C4), 2.40 (s, 6H, 2xCH_3_), 2.46 (s, 3H, CH_3_ at C8), 3.96 (s, 2H, CH_2_), 5.08 (s, 2H, OCH_2_), 5.66 (s, 1H, CH pyrazole), 6.87 (d, 1H, *J*=8.9 Hz, H-6 Ar), 7.17 (t, 1H, H-4′ Ar), 7.23 (t, 2H, H-3′,5′ Ar), 7.27 (d, 2H, *J* = 7.4 Hz, H-2′,6′ Ar), 7.57 (d, 1H, *J*=8.9 Hz, H-5 Ar). MS m/z %: 416 (M^+^) 1.91%. Anal. Calcd. for C_25_H_24_N_2_O_4_ (416.47): Calc.: C, 72.10; H, 5.81; N, 6.73. Found: C, 71.89; H, 5.89; N, 7.02.

### 2.2 Biological Activity

#### 2.2.1 Antitumor Screening

The synthesized compounds were subjected to the NCI’s disease-oriented human cell lines screening assay to be evaluated for their *in-vitro* antitumor activity. The anticancer assays were performed in accordance with the protocol of the Drug Evaluation Branch, NCI, Bethesda [14-18].

Under a sterile condition, the human tumor cell lines of the cancer screening panel were grown in RPMI 1640 medium containing 5% fetal bovine serum and 2 mM L-glutamine. For a typical screening experiment, the cells were inoculated into 96 well microtiter plates in 100 μL at plating densities ranging from 5,000 to 40,000 cells/well depending on the doubling time of individual cell lines. After cell inoculation, the microtiter plates were incubated at 37° C, 5% CO_2_, 95% air and 100% relative humidity for 24 h prior to the addition of experimental drugs.

After 24 h, two plates of each cell line were fixed *in situ* with trichloroacetic acid (TCA), to represent a measurement of the cell population for each cell line at the time of drug addition (*Tz*). Experimental drugs were solubilized in dimethylsulfoxide (DMSO) at 400-fold achieving the desired final maximum test concentration and stored frozen prior to use. At the time of drug addition, an aliquot of frozen concentrate was thawed and diluted to twice the desired final test concentration (10^-5^ M) with complete medium containing 50 μg/mL gentamicin. Aliquots of 100 μL of these drug dilutions were added to the appropriate microtiter wells already containing 100 μL of medium, resulting in the required final drug concentrations.

Following drug addition, the plates were incubated for an additional 48 h at 37°C, 5% CO_2_, 95% air, and 100% relative humidity. For adherent cells, the assay was terminated by the addition of cold TCA. Cells were fixed *in situ* by gently adding 50 μL of cold 50% (w/v) TCA (final concentration, 10% TCA) and incubated for 60 min. at 4 °C. The supernatant was discarded, and the plates were washed five times with tap water and air dried. Sulforhodamine B (SRB) solution (100 μL) at 0.4% (w/v) in 1% acetic acid was added to each well, and plates were incubated for 10 min at room temperature. After staining, unbound dye was removed by washing five times with 1% acetic acid and the plates were air dried. Bound stain was subsequently solubilized with 10 mM trizma base, and the absorbance was read on an automated plate reader at a wavelength of 515 nm [19].

Using the seven absorbance measurements [time zero, (*Tz*), control growth, (*C*), and test growth in the presence of drug at the 10^-5^ M concentration level (*Ti*)], the percentage growth was calculated at each of the drug concentrations levels. Percentage growth inhibition was calculated as:

[(*Ti* − *Tz*)/(*C* − *Tz*)] x 100 for concentrations for which Ti ≥ Tz

[(*Ti* − *Tz*)/*Tz*] x 100 for concentrations for which Ti < Tz.

##### 2.2.1.1 The Mean Graph

Mean graph is the mean presenting the in vitro test results to emphasize differential effects of test compounds on various human tumor cell lines. It plots the growth relative to no drug control and relative to time zero number of cells. The mean is the average of growth across the tested cell lines, while delta is the maximum difference from the mean.

## 3 RESULTS AND DISCUSSION

### 3.1 Chemistry

**Scheme (1):**
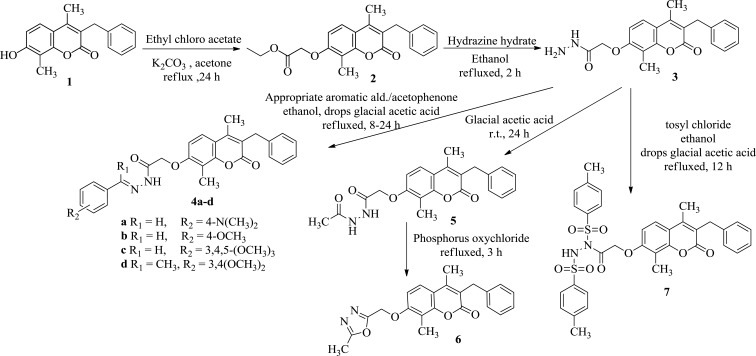
Synthesis of Sciffs like compounds 4a-d,acetohydrazide 5,oxadiazole 6 and methylphenylsulphonyl derivative 7.

**Scheme (2):**
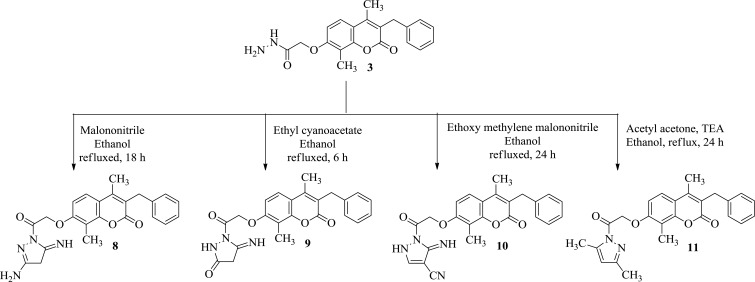
Synthesis of substituted pyrazoles 8-11.

The intermediates **2**, **3** and the target compounds **4(a-d)-11** were synthesized as depicted in (Scheme **1** and **2**).

The starting compound **1** was prepared as reported in literature [13]. Benzylidene acetohydrazides derivatives were prepared by refluxing acid hydrazide with appropriate aromatic aldehyde or acetophenone in ethanol/ glacial acetic acid, and this method was adopted for the synthesis of compounds **4a-d.** The structures of **4a-d** were confirmed with elemental analyses and spectral data. IR spectra elicited a band at 3460-3188 cm^-1^ corresponding to NH group. ^1^H NMR of **4a-c** displayed a singlet at 7.89-8.22 ppm corresponding to azo methane proton and another singlet at 8.50-11.57 ppm assigned to NH. While compound **4d** revealed a singlet signal at 2.53 ppm corresponding to the N=CCH_3_ protons and two singlet signals at 3.81 and 3.84 ppm corresponding to the added two OCH_3_ protons. Finally, MS spectra revealed their molecular ion peaks. Compound **5** was obtained by stirring a mixture of acid hydrazide **3** with glacial acetic acid at room temperature for 24 h. The structure of **5** was elucidated by the elemental analysis and spectral data. IR spectrum showed bands at 3446, 3238 cm^-1^ corresponding to 2 NH groups. ^1^H NMR spectrum revealed a singlet signal at δ = 1.88 ppm corresponding to COCH_3_ proton and 2 singlet signals at δ = 9.89 and 10.04 ppm corresponding to 2 NH groups. The MS spectrum revealed its molecular ion peak at 394. Synthesis of substituted oxadiazoles can be achieved through one pot reaction of three components, phosphorus oxychloride, acid hydrazide and acid derivatives [20]. Another method involves cyclization of acetohydrazide derivatives using phosphorus oxychloride in dioxane [21]. In the present work, the later method was followed to prepare **6** in good yield. Compound **6** was confirmed through the elemental analysis and spectral data. IR spectrum showed disappearance of bands corresponding to 2 NH groups and only one band at 1691 cm^-1^ corresponding to benzopyrone C=O group. ^1^H NMR spectrum revealed disappearance of any signals corresponding to the 2 NH protons. MS spectrum showed its molecular ion peak at *m/z* 367. Reaction of acid hydrazide derivatives with acid chloride as tosyl chloride led to the formation of benzene sulfone hydrazide derivatives, this reaction was reported to be performed in glacial acetic acid at room temperature for 24 h [22]. The structure of **7** was deduced as disubstituted derivative by the elemental analysis and spectral data. IR spectrum showed a band at 3446 cm^-1^ corresponding to NH group and bands at 1354, 1188 cm^-1^ corresponding to SO_2_ groups. ^1^H NMR spectrum showed a singlet signal at δ = 2.29 ppm corresponding to two *p*-CH_3_ protons, a double of doublet at δ = 7.12 and 7.50 ppm corresponding to 3ʺ, 5ʺ and 2ʺ, 6ʺ protons of the two *p*-methylbenzenesulphone moieties, respectively. The MS spectrum of **7** revealed the molecular ion peak at 660.

Refluxing acid hydrazide **3** with many substituted carbonitrile derivatives as malononitrile, ethyl cyanoacetate and ethoxy methylene malononitrile was reported to yield the corresponding amino pyrazoles **8**, pyrazolone **9** and pyrazole carbonitriles **10**, respectively. The target compounds **8**, **9**, and **10** were synthesized through refluxing acid hydrazide derivative **3** with appropriate carbonitrile compound in ethanol (Scheme **2**).

The IR spectrum of **8** showed bands at 3504, 3448 and 3404 cm^-1^ assigned to NH_2_ and NH groups. ^1^H NMR spectra of **8** displayed three singlet signals at 1.88 and 4.65 and 9.93 ppm corresponding to CH_2_ pyrazoline, NH and NH_2_, respectively. ^1^H NMR spectra of **9** revealed two singlet signals at δ = 1.87 and 9.67 ppm corresponding to CH_2_ pyrazolone and two NH protons, respectively. IR spectrum of **10** revealed a characteristic band at 2196 cm^-1^ corresponding to the added cyano group. Finally, MS spectra revealed the molecular ion peaks of the titled compounds. Condensation of acid hydrazide with acetyl acetone in ethanol containing triethylamine afforded the corresponding dimethyl pyrazoles **11** (Scheme **2**). The structure of **11** was deduced from microanalytical and spectral data. ^1^H NMR spectra showed new two singlet signals at 2.40 ppm assigned to six protons of the two CH_3_ substituting the pyrazole ring and at 5.66 ppm corresponding to the CH of pyrazole, finally **11** revealed the molecular ion peak at 416.

### 3.2 Antitumor Screening

#### 3.2.1 Preliminary *In Vitro* Antitumor Screening

Newly synthesized compounds (**4a-d, 5, 6, 7, 8, 9, 10 and 11)** were selected by National Cancer Institute (NCI) Developmental Therapeutic Program (www.dtp.nci.nih.gov), Bethesda, MD, U.S.A. The synthesized compounds were subjected to the NCI’s disease-oriented human cell lines screening assay to be evaluated for their *in vitro* antitumor activity. The anticancer assays were performed in accordance with the protocol of the Drug Evaluation Branch, NCI, Bethesda [14-18]. A single dose (10 µM) of the test compounds was used in the full NCI 60 cell line panel assay. A 48 h drug exposure protocol was used and sulforhodamine B (SRB) protein assay was applied to estimate the cell viability and growth [19]. The results were reported as mean graph of the percent growth of the treated cells and presented as percentage growth inhibition (GI %). The obtained results of the tested benzopyrone analogues showed distinctive potential pattern of selectivity, as well as broad-spectrum antitumor activity (Table **1**).

Regarding the activity towards individual cell lines, Schiffs like compounds **4a**-**d** showed overall moderate activity with **4a-c** having a better activity with mean GI values of 6.17, 10.84 and 7.99% for **4a**, **4b** and **4c**, respectively compared to **4d**. Regarding the activity of each compound, the benzylidene derivative **4a** achieved only a moderate effect upon leukemia HL-60 (TB) with GI value of 30.93% and a weak activity over the leukemia subpanels CCRF-CEM and SR with GI values of 19.49 and 25.42%, respectively. A noticeable effect was achieved upon many non-small cell lung cancer subpanels with cell line NCI-H226 having the greatest effect of GI value of 46.05% while NCI-H23, NCI-H332M and NCI-H522 was inhibited by values of 15.35, 22.25 and 19.71%, respectively. An overall weak activity was revealed over the other subpanels tested with GI values of 15.28, 11.02, 10.69, 21.11, 12.95, 15.27, 10.23, 28.13, 11.26, 10.65 and 13.56 for CNS cancer subpanels SF-268, SNB-19, U251, melanoma subpanels LOXIMVI-MALME-3M, UACC-62, ovarian cancer OVCAR-8, renal cancer A498, SN12C, UO-31, prostate cancer PC-3 and finally breast cancer MCF-7 and MDA-MB-231/ATCC, respectively. The 4-methoxybenzylidene derivative **4b** revealed similar activity to that of the *N*,*N*-dimethylaminobenzylidene derivative **4a** with the highest effect achieved over the CNS cancer subpanel SNB-75 with GI value of 44.61%. The other cell lines tested were inhibited with weak to moderate effect with GI values of 15.78, 23.39, 13.84 and 20.78% for leukemia cell lines CCRF-CEM, HL-60 (TB), MOLT-4 and RPMI-8226, respectively. While the non-small cell lung cancer subpanels A549/ATCC, HOP-92, NCI-H226, NCI-H23, NCI-H322M and NCI-H522 with GI values of 17.44, 14.88, 12.03, 13.99 and 22.18%, respectively. Colon cancer subpanels HCT-116 and KM12 were inhibited by GI values of 13.60 and 10.00%, respectively. A moderate effect was achieved upon melanoma subpanel UACC-257 with GI value of 21.41%, the renal cancer RXF393 with GI value of 18.22% and prostate cancer cell line PC-3 was inhibited by 21.06%. Finally, GI values of 21.06 and 19.15% were achieved over breast cancer subpanels MDA-MB-231/ATCC and MDA-MB-468, respectively. The trimethoxybenzylidene **4c** inhibited leukemia subpanels RPMI-8226 and SR with GI values of 24.64 and 29.69%. A good effect was shown upon the non-small cell lung cancer subpanel NCI-H522 with GI value of 31.83%. GI values of 34.48 and 30.04% were achieved over renal cancer UO-31 and breast subpanel MDA-MB-468, respectively. The dimethoxyphenylethylidene derivative **4d** showed a weak activity over a single cell line, the renal cancer UO-31 with GI value of 15.52%.

The acetohydrazide derivative **5**, oxadiazole **6** and bis-methylphenylsulphonyl derivative **7** showed a similar inhibitory pattern. The three compounds revealed only a weak effect upon leukemia subpanel RPMI-8226 with GI values of 15.42, 12.64 and 11.32% for **5**, **6** and **7**, respectively. Also the non-small cell lung cancer cell line NCI-H522 was inhibited by 13.08, 10.75 and 15.72% for **5**, **6** and **7**, respectively. Finally, **5**, **6** and **7** exhibited a weak activity over renal cancer UO-31 with GI values of 21.08, 11.03 and 21.24%, respectively.

The 3-amino-5-imino-4,5-dihydropyrazol **8** derivative, 5-iminopyrazolidin-3-one derivative **9** and 5-imino-2,5-dihydropyrazole-4-carbonitrile **10** revealed no noticable effect, all three derivatives shared activity towards non-small cell lung cancer subpanel NCI-H522 with GI values 22.46, 21.43 and 20.63%, respectively, they also shared activity towards renal cancer UO-31 with GI values of 29.22, 35.81 and 24.45%, respectively. **8** also possessed weak activity towards breast cancer T-47D with GI value of 29.36% where it showed the highest mean GI among the three derivatives of value 3.66% compared to mean GI value of 2.08 and 0.95% corresponding to **9** and **10**, respectively. Compound **11** did not exhibit considerable activity as GI values were 12.22 and 15.14% against non-small cell lung cancer NCI-H522 and renal cancer UO-31, respectively.

## CONCLUSION

New benzopyrone derivatives were prepared in this study. All compounds were selected by National Cancer Institute (NCI), Bethesda, and evaluated for their *in vitro* anticancer activity in the full NCI 60 cell lines panel assay by a single dose test (10 µM). Substituted benzylidene derivatives **4a, b** and **c** had the best activity with mean GI values of 6.17, 10.84 and 7.99%, respectively. Results revealed that, Schiff’s like compounds of benzopyrone scaffold with substituted benzylidene derivatives **4a-c** had overall good effect. However, Schiff’s like compounds comprised of disubstituted phenylethylidene derivative **4d** had no significant effect. In addition, aetohydrazide derivatives **5** and **7** or hybrids with oxadiazole **6** or substituted pyrazoles **8**-**11** had a weak or no significant effect.

## Figures and Tables

**Table 1:** Growth inhibition percent of tested compounds against 60 different cell lines.

**Panel/Cell Line**	**-**	**Test compounds and growth inhibition percent of cell line**									
	**4a**	**4b**	**4c**	**4d**	**5**	**6**	**7**	**8**	**9**	**10**	**11**
**Leukemia**											
CCRF-CEM	19.49	15.78	-	-	-	-	-	-	-	-	-
HL-60 (TB)	**30.93**	23.39	-	-	-	-	-	-	-	-	-
K-562	-	-	-	-	-	-	-	-	-	-	-
MOLT-4	-	-	14.21	-	-	-	-	-	-	-	-
RPMI-8226	-	13.84	24.64	-	-	-	-	11.51	-	-	-
SR	25.42	20.78	29.69	**-**	15.42	12.64	11.32	17.70	-	-	-
**Non-Small Cell Lung Cancer**											
A549/ATCC	-	20.29	17.33	-	-	-	-	10.33	-	**-**	-
EKVX	-	-	-	-	-	-	-	-	-	-	-
HOP-92	**46.05**	17.44	11.58	**-**	-	-	-	14.05	15.45	10.92	-
NCI-H226	15.35	14.88	17.63	-	-	-	-	-	12.25	-	-
NCI-H23	-	12.03	10.10	-	-	-	-	-	-	-	-
NCI-H322M	22.25	13.99	12.49	-	-	-	-	20.33	20.19	-	-
NCI-H460	-	-	-	-	-	-	-	-	-	-	-
NCI-H522	19.71	22.18	**31.83**	**-**	13.08	10.75	15.72	22.46	21.43	20.63	12.22
**Colon Cancer**											
COLO 205	-	-	-	-	-	-	-	-	-	-	-
HCC-2998	-	-	-	-	-	-	-	-	-	-	-
HCT-116	-	13.60	-	-	-	-	-	-	-	-	-
HCT-15	-	-	-	-	-	-	-	-	-	-	-
HT29	-	-	-	-	-	-	**-**	-	-	-	-
KM12	-	10.00	-	-	-	-	-	-	-	-	-
SW-620	-	-	-	-	-	-	-	-	-	-	-
**CNS Cancer**											
SF-268	15.28	11.01	-	-	-	-	-	-	-	-	-
SF-295	-	13.86	-	-	-	-	-	-	-	-	-
SF-539	-	12.74	-	-	-	-	-	-	-	-	-
SNB-19	11.02	-	10.74	-	-	-	-	-	-	-	-
SNB-75	**-**	**44.61**	13.03	-	-	-	-	-	-	19.00	**-**
U251	10.69	10.79	-	-	-	-	-	-	-	-	-
**Melanoma**											
LOX IMVI	21.11	-	11.55	-	-	-	-	-	-	-	-
MALME-3M	-	16.27	-	-	-	-	-	-	-	-	-
M14	-	-	-	-	-	-	-	-	-	-	-
MDA-MB-435	-	-	-	-	-	-	-	-	-	-	-
SK-MEL-2	-	-	-	-	-	-	-	-	-	-	-
SK-MEL-28	-	-	-	-	-	-	-	-	-	-	-
SK-MEL-5	-	-	12.50	-	-	-	-	-	-	-	-
UACC-257	12.95	21.41	12.10	-	-	-	-	-	-	-	-
UACC-62	-	-	23.91	-	-	-	-	-	-	-	-
**Ovarian cancer**											
IGROV1	-	-	10.63	-	-	-	-	-	16.75	-	-
OVCAR-3	-	10.81	-	-	-	-	-	-	-	-	-
OVCAR-4	-	15.15	-	**-**	-	-	-	-	-	-	-
OVCAR-5	-	-	-	-	-	-	-	-	-	-	-
OVCAR-8	-	15.93	-	-	-	-	-	-	-	-	-
NCI/ADR-RES	-	-	-	-	-	-	-	-	-	-	-
SK-OV-3	-	-	-	-	-	-	-	-	-	-	-
**Renal cancer**											
786-0	-	-	-	-	-	-	-	-	-	-	-
A498	15.27	14.27	10.90	-	-	-	-	-	-	-	-
ACHN	-		-	-	-	-	-	-	-	-	-
RXF 393	-	18.22	-	-	-	-	-	-	-	-	-
SN12C	10.23	-	-	-	-	-	-	-	-	-	-
TK-10	-	-	-	-	-	-	-	-	-	-	-
UO-31	28.13	-	**34.48**	15.52	21.08	11.03	21.24	29.22	35.81	24.45	15.14
**Prostate cancer**											
PC-3	11.26	18.85	18.92	-	-	-	-	-	14.89	**11.04**	-
DU-145	-	-	-	-	-	-	-	-	-	-	-
**Breast cancer**											
MCF7	10.65	10.50	14.72	**-**	-	-	-	10.44	-	10.28	-
MDA-MB-231/ATCC	13.56	21.06	17.25	-	-	-	-	-	-	-	-
HS 578T	-	11.68	10.07	-	-	-	-	-	-	-	-
BT-549	-	-	-	-	-	-	-	13.86	-	-	-
T-47D	-	19.15	**30.04**	**-**	-	-	-	29.36	-	18.11	**-**
MDA-MB-468	-	-	-	-	-	-	-	-	-	-	-
**Mean GI%**	**6.17**	**10.84**	**7.99**	**0.00**	**0.00**	**0.00**	**0.00**	**3.66**	**2.08**	**0.95**	**0.00**

(-, GI <10%)
